# Heteroplasmy in the complete chicken mitochondrial genome

**DOI:** 10.1371/journal.pone.0224677

**Published:** 2019-11-08

**Authors:** Yanqun Huang, Weiwei Lu, Jiefei Ji, Xiangli Zhang, Pengfei Zhang, Wen Chen

**Affiliations:** College of Livestock Husbandry and Veterinary Engineering, Henan Agricultural University, Zhengzhou, Henan, China; Friedrich-Loeffler-Institute, GERMANY

## Abstract

Chicken mitochondrial DNA is a circular molecule comprising ~16.8 kb. In this study, we used next-generation sequencing to investigate mitochondrial heteroplasmy in the whole chicken mitochondrial genome. Based on heteroplasmic detection thresholds at the 0.5% level, 178 cases of heteroplasmy were identified in the chicken mitochondrial genome, where 83% were due to nucleotide transitions. D-loop regionwas hot spot region for mtDNA heteroplasmy in the chicken since 130 cases of heteroplasmy were located in these regions. Heteroplasmy varied among intraindividual tissues with allele-specific, position-specific, and tissue-specific features. Skeletal muscle had the highest abundance of heteroplasmy. Cases of heteroplasmy at *mt*.*G8682A* and *mt*.*G16121A* were validated by PCR-restriction fragment length polymorphism analysis, which showed that both had low ratios of heteroplasmy occurrence in five natural breeds. Polymorphic sites were easy to distinguish. Based on NGS data for crureus tissues, mitochondrial mutation/heteroplasmy exhibited clear maternal inheritance features at the whole mitochondrial genomic level. Further investigations of the heterogeneity of the *mt*.*A5694T* and *mt*.*T5718G* transitions between generations using pyrosequencing based on pedigree information indicated that the degree of heteroplasmy and the occurrence ratio of heteroplasmy decreased greatly from the F0 to F1 generations in the *mt*.*A5694T* and *mt*.*T5718G* site. Thus, the intergenerational transmission of heteroplasmy in chicken mtDNA exhibited a rapid shift toward homoplasmy within a single generation. Our findings indicate that heteroplasmy is a widespread phenomenon in chicken mitochondrial genome, in which most sites exhibit low heteroplasmy and the allele frequency at heteroplasmic sites changes significantly during transmission events. It suggests that heteroplasmy may be under negative selection to some degree in the chicken.

## Introduction

The chicken mitochondrial genome is a circular DNA molecule comprising about 16.8 kb, which encodes 13 proteins, two *rRNAs*, and 22 *tRNA*s in the same manner as other types of vertebrate mitochondrial DNA (mtDNA) [1]. There are hundreds to thousands of mtDNA copies per cell and mtDNA has a very high mutation rate. Heteroplasmy refers to the presence of more than one mtDNA variant within a cell, tissue, or individual, and it is not as rare as previously considered. Many human mutations exist in a heteroplasmic state (https://www.mitomap.org/MITOMAP) and the extent of some disease symptoms can vary according to the proportion of the deleterious allele [2, 3].

Heteroplasmy has been detected using various approaches, including Sanger capillary sequencing [4], cleaved amplification polymorphism sequence-tagged sites (PCR-restriction fragment length polymorphism; PCR-RFLP) [5], and pyrosequencing [6]. However, these approaches are site limited. In addition, sanger sequencing does not provide quantitative information and it is not adequately sensitive for detecting mutant heteroplasmy below 15% [4]. The detection limit for PCR-RFLP based on ethidium bromide gel analysis is 5–10% [5].

Recently, next-generation sequencing (NGS sequencing) has been used to study human mitochondrial heteroplasmy and several computational approaches have been developed for heteroplasmy detection [7–11]. Deep sampling with the NGS approach provides a simple, high-throughput, and cost-effective platform for efficiently detecting and quantifying mitochondrial heteroplasmy in whole mitochondrial genomes [9]. Heteroplasmic variants can be routinely detected by NGS down to a component ratio of 1:250 and they can be readily detected down to 1:1000 (0.1%) with expanded coverage [12]. The heteroplasmy signals become increasingly difficult to distinguish from sequencing errors as the heteroplasmy level decreases to approximately 0.1% [13]. Giuliani et al. detected 119 heteroplasmic positions with a minor allele frequency (MAF) ≥ 0.2%, and found that low level cases of heteroplasmy were transmitted and maintained within families until extreme ages [11], thereby demonstrating that heteroplasmy is not as rare as previously considered, and it is emerging as an important component of eukaryotic genetic diversity [7, 10].

Previous research into heteroplasmy has focused on humans, whereas few studies have considered heteroplasmy in other animals such as chicken [14], pig [15], dog[16] and cattle [17]. It is increasingly clear that heteroplasmy plays an important biological role in poultry. Lu et al. reported two cases of heteroplasmy in the chicken mitochondrial ND2 gene, which is associated with the pectoral muscle fat content [14]. There have been no previous reports of heteroplasmy in the complete mitochondrial genome in poultry. In this study, we investigated the distribution of heteroplasmy in chicken mtDNA and heteroplasmic differences among multiple tissues at the whole mitochondrial genomic level by NGS sequencing. We also focused on the examples of heteroplasmy at *mt*.*G8682A* and *mt*.*G16121A* in five natural breeds and analyzed the transmission of heteroplasmy at *mt*.*A5694T* and *mt*.*T5718G* based on pedigree information for a constructed population. The chicken is an important animal model. Our findings also provide novel insights into human mitochondrial heteroplasmy.

## Materials and methods

### Collection of blood samples from different breeds

Anticoagulant blood samples were collected from laying chickens at 45 weeks old comprising White Leghorn (LH, n = 10), White Plymouth Rock (PR, n = 10), Silky (SK, n = 10), Beijing You chicken (BY, n = 10), and Tibetan chicken (TB, n = 10), which were provided by the Henan Poultry Germplasm Resources Innovation Engineering Center. The birds were raised in cages and given free access to food and water. The diets were formulated according to the nutritional standards for laying chickens (NRC, 1994). Chicken tissue/blood DNA was extracted according to the phenol–chloroform extraction method.

### Construction of a heteroplasmic population for NGS sequencing and heteroplasmic transmission

Fertilized eggs were collected from the following mating populations (45 weeks old): Silky♂×Silky♀ (SS), Rhode Island Red♂×Rhode Island Red♀ (RR), Silky♂× Rhode Island Red♀ (SR), Rhode Island Red♂×Silky♀ (RS), and Silky♂×Gushi Chicken♀ (SG), and pedigree hatching was conducted (the populations were designated as heteroplasmic populations). Samples of anticoagulant-treated blood were taken from the parent population (F0 generation) after collecting the fertilized eggs from related individuals. The young chicks (F1 generation) were tagged and raised in cages under conventional conditions, where food and water were provided *ad libitum*. At 1, 30, and 60 days of age, F1 generation chicks were randomly selected from different groups and sacrificed. Anticoagulant-treated blood samples and multiple tissues were collected for analysis. In addition, at least 15 tissues were collected from one RR chicken and mixed to prepare homogenates at 1 and 60 days of age. The collected tissue samples were snap frozen in nitrogen and stored at below –80°C until NGS sequencing. The blood samples obtained from F0 and F1 individuals were used for detecting heteroplasmic transmission between the parents and offspring. Tissue/blood DNA samples were extracted according to the phenol–chloroform extraction method. All procedures were approved by the Animal Care and Use Committee of Henan Agricultural University (Zhengzhou, China).

### Long-range PCR (LG-PCR)

LG-PCR was conducted as described by Zhang et al. [18]. Briefly, a set of back-to-back primers (designated as PM primers; Table 1) were designed based on the NC_001323.1 sequence for amplifying the whole mitochondrial genome. The product comprised approximately 16.8 kb. We performed PCR amplification with about 15–50 ng tissue/blood DNA as the template in a 50-μL PCR system, using Lamp^TM^ DNA Polymerase with Mg^2+^ plus buffer (Vazyme Biotech Co. Ltd) under the following conditions: initial incubation at 94°C for 2 min, followed by 30 PCR cycles with denaturation at 94°C for 30s, and annealing and extension at 68°C for 10 min, before one final extension cycle at 72°C for 7 min and holding at 4°C. Next, 3 μL of the PCR products were subjected to electrophoresis on 1.5% agarose gels. The LG-PCR products were purified by DNA gel extraction kit (Generay, Shanghai, China) and then conducted Illumina HiSeq Sequencing.

**Table 1:** The used primers.

Name	Primer sequence (5'–3')	purpose	Length (bp)	Tm (°C)	Enzyme
PW	F:5'GTTGCGTCCTATCCTAGTCCTCTCG3' R: 5' GCAGGTGTAGTCCAGGCTTCACTT3'	whole mtDNA	16775	68	--
P8682	F: 5'AAACACCGTAGATGCCCAAG3' R: 5' TAGGGGGAGGTCTGTTGTTG3'	*mt*.*G8682A*	232	60	*Hinf*I
P16121	F:5' TCCACCCTTCTTAGAGTATCAG 3' R: 5' TGGGTGAGGTTTGTTGTTAG 3''	*mt*.*G16121A*	176	57	*Hinf*I
PND2	F:5′ CCTCCTCCTAACATCACAGTCTCTTAA 3′ R:5′ AGAAGGCTAGGATTTTTCGTGTTTGT 3′	*mt*.*A5694T mt*.*T5718G*	122	60	

Note: The reference sequence is NC_001323.1.

### DNA template library preparation for Illumina indexed sequencing

#### Amplicon sequencing by Illumina HiSeq

Paired-end index libraries were constructed according to the manufacturer’s instructions (NEBNext® Ultra^™^ DNA Library Prep Kit for Illumina®) with minor modifications. Briefly, the LG-PCR products were randomly fragmented into sizes <400 bp by sonication (Diagenode Bioruptor UCD-200). The fragments were treated with End Prep Enzyme Mix. Size selection was then performed for the adaptor-ligated DNA using AxyPrep Mag PCR Clean-up (Axygen), and fragments of ~400 bp (with approximate insert sizes of 250 bp) were recovered. Each sample was amplified by PCR for eight cycles using P5 and P7 primers. The PCR products were cleaned using AxyPrep Mag PCR Clean-up (Axygen), validated with an Agilent 2100 Bioanalyzer (Agilent Technologies), and quantified by Qubit and real-time PCR (Applied Biosystems). Libraries with different indexes were multiplexed and loaded onto an Illumina HiSeq instrument according to the manufacturer’s instructions (Illumina, San Diego, CA, USA). Sequencing was performed using a 2 × 100 paired-end (PE) configuration. Image analysis and base calling were conducted with HiSeq Control Software (HCS) + OLB + GAPipeline-1.6 (Illumina) on the HiSeq instrument. The sequences were processed and analyzed by GENEWIZ using NGSQCToolkit (v2.3).

#### Quality control for reads

Dirty reads were removed using NGSQCToolkit (v2.3) software and the following quality control processes were conducted: (1) removal of primers and adaptors; (2) removal of 3' end bases with quality values below 30 and ambiguous bases; (3) retaining over 75% of the reads with quality values above 30; and (4) removing reads with N over 10%. The clean reads were used for the subsequent analyses.

#### Identification of heteroplasmic sites

Burrows-Wheeler Aligner (BWA) mapper[19] (version 0.7.12, http://bio-bwa.sourceforge.net/) was used to initially map the reads (NC_001323.1 as the reference sequence). SAMtools[20] (version 1.1, http://samtools.sourceforge.net/) was employed for processing the generated Sequence Alignment/Map (SAM) data sets and removing duplicate reads. Statistical analyses of the base distribution for each locus were performed with the pileup2base script (pileup2 baseindel.pl, https://github.com/riverlee/pileup2base). Variant heteroplasmy was expressed as the alternative allele frequency (AAF) calculated as: base heteroplasmy (AAF%) = alternative allele (forward + reverse)/total coverage of all alleles C, G, T, and A (forward + reverse) × 100. We defined the heteroplasmic detection threshold as a MAF of at least 0.5%.

In total, 30 of 48 samples lacked sufficient clean reads and they were omitted from subsequent analyses. The average sequencing coverage for the remaining individuals was ∼7,800×(range:26.5–6,5000×). The data was deposited in the Sequence Read Archive (SRA) at the National Center for Biotechnology Information (NCBI) under accession numbers (SRR10225379-SRR10225396). We focused on cases of heteroplasmy involving single base substitutions. The deep coverage allowed the detection of very low mutation heteroplasmy at any of the 16,775 nucleotide positions. It has been shown that the analytic sensitivity of heteroplasmy detection is correlated with the coverage depth. We routinely obtained a coverage depth of about 10,000–20,000 fold for the mitochondrial genome.

#### PCR-RFLP

The two heteroplasmic sites comprising *mt*.*G8682A* (D118H) and *mt*.*G16121A* (referenced to NC_001323.1) exhibited different heteroplasmic levels according to NGS sequencing. Both the changes of *mt*. *8682G*→*A* and *mt*. *16121A*→*G* could lead to the loss of the *Hinf*1 enzyme site. PCR-RFLP was used to identify the heteroplasmy of *mt*.*G8682A* and *mt*.*G16121A* in the NGS samples and the blood DNA samples of five breeds (LH, PR, SK, BY, and TB). Briefly, the PCR products amplified with primer sets P8682 and P16121 (Table 1) were digested with the *Hinf*1 enzyme according to the manufacturer’s instructions (Fermentas, MBI) and the cleavage products were separated by 3% agarose electrophoresis. The heteroplasmic ratio was expressed as the certain allelic ratio based on the grey value detected using an Automatic Gel Imaging and Analysis System (ChampGel 5000, Beijing Sage Creation Science Co., Ltd, Beijing, China). For example, the ratio of allele *mt*.*8682A* for *mt*.*G8682A* was the grey value of: base A/grey value of base (A + G) × 100%.

### Pyrosequencing

Pyrosequencing was conducted with blood DNA samples to detect the heteroplasmy of *mt*.*A5694T* and *mt*.*T5718G* in the F0 and F1 generations of the constructed heteroplasmic population. Briefly, fragments containing *mt*.*A5694T* and *mt*.*T5718G* mutations (referenced to NC_001323.1 in the *ND2* gene) were amplified with PND2 primers (Table 1). The PCR products were purified and subjected to clone sequencing. *mt*.*A5694T* and *mt*.*T5718G* were in strong linkage disequilibrium. The clones with the 5694A-5718T and 5694T-5718G haplotypes were selected as the positive controls for pyrosequencing. A pyrosequencing primer (5′-CCATTCAGCCTCCGA-3′) was used as the sequencing primer and all of the steps were performed according to the manufacturer’s protocol. The relative ratios of the two alleles in the *mt*.*A5694T* and *mt*.*T5718G* sites were scored. All samples were analyzed in triplicate.

## Results

### Heteroplasmy information for 18 NGS samples

**Fig 1:**
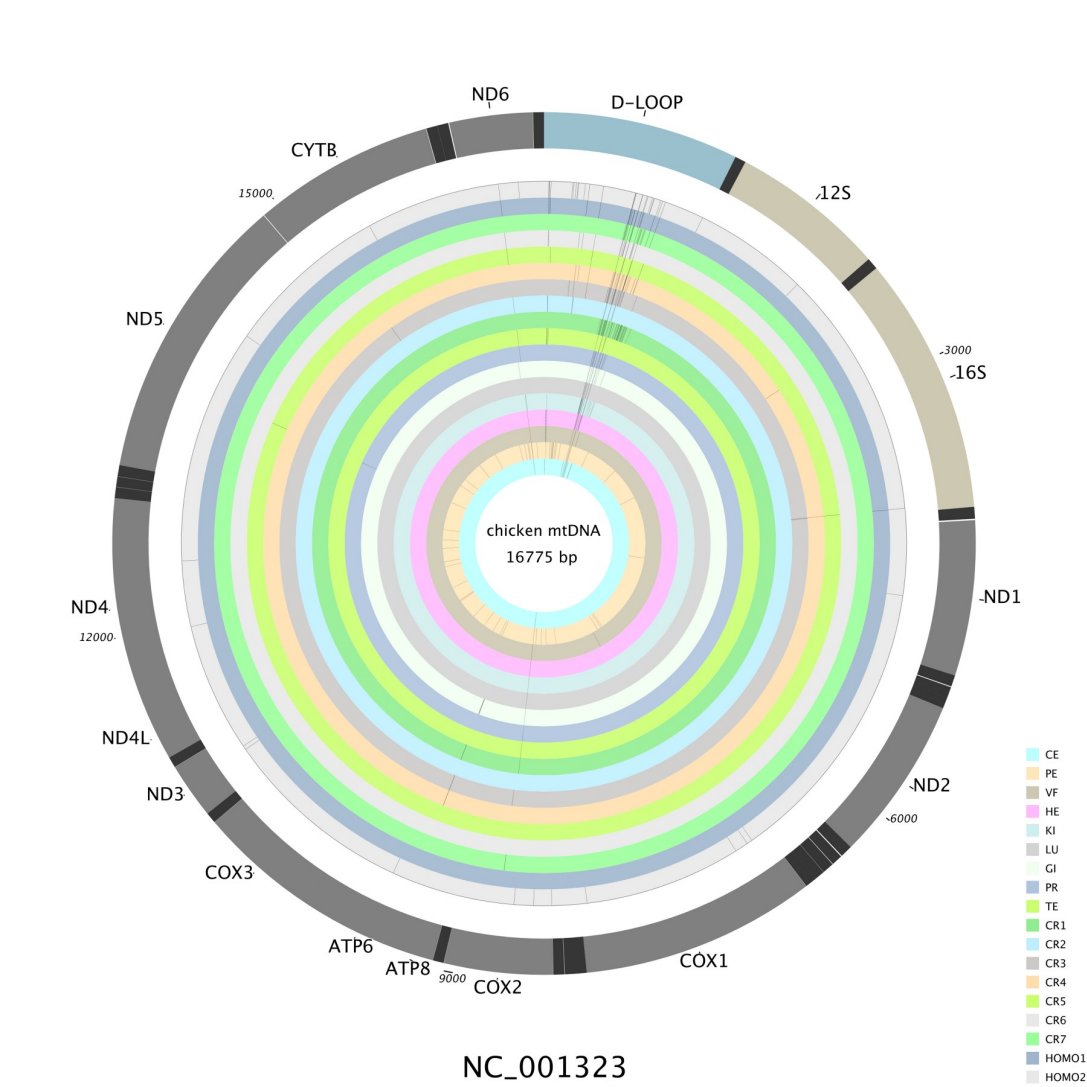
Distribution of heteroplasmies across tissues (intra-individual) and all individuals in the chicken mtDNA genome. It was ordered as legend from the innermost to outermost ring. Here the CR samples from different individuals were named as CR1-CR7, the corresponding individuals and populations were presented in S1 Table. Intra-individual tissues included CE, PE, VF, HE, KI, LU, GI, PR, TE, CR1. Homo1and Homo2 is two mixed samples. Abbreviations: CE, cerebrum; PE, pectoral; VF, visceral fat; HE, heart; KI, kidney; LU, lung; GI, gizzard; PR, proventriculus; TE, testis; CR, crureus; Homo, mixed tissue.

NGS sequencing with the LG-PCR products amplified using the set of back-to-back primers (S1 Fig) allowed us to examine each nucleotide in the entire 16.8 kb mitochondrial genome, thereby providing uniform coverage and sufficient depth for quantifying heteroplasmy. Eighteen samples yielded sufficient data and 30 samples were discarded because of their low quality data. Based on an average coverage of ~7,800× (S2 Fig), we used stringent criteria (Materials and Methods) to identify 178 cases of heteroplasmy where MAF ≥0.5% (Fig 1). The cases of heteroplasmy were distributed across 168 positions in the chicken mtDNA genome, with MAF varying from 1 to 40.3% (Table 2, S1 Table).

**Table 2:** The distribution of hereoplasmic sites among 18 samples (detected by next-generation sequencing).

Mating^a^		RS1										SG1	RR1	RR2	RR3	SR1	SR2	Total^d^	RR4	RR5	Total^e^
age(d)		60										60	1	30	60	60	60		1	60	
Tissue^b^	Total^c^	CE	PE	VF	HE	KI	LU	GI	PR	TE	CR1	CR2	CR3	CR4	CR5	CR6	CR7	CR	Homo1	Homo2	
MAF≥0.5%	137	12	53	10	17	25	10	17	20	11	48	11	30	28	12	18	20	93	20	45	178
MAF≥1%	34	3	10	4	7	7	5	6	7	4	22	7	11	9	4	4	8	36	10	9	53
MAF≥5%	7	2	2	3	2	3	3	2	3	2	7	2	2	3	2	2	3	7	2	2	7
MAF≥10%	5	2	2	2	2	3	3	1	3	2	4	1	2	2	2	2	3	5	2	2	5
AAF≥50%	8	8	8	8	8	8	8	8	8	8	8	9	34	34	34	34	34	43	34	34	43
TSS	114	5	44	3	7	8	1	5	7	2	32										
UN	0	4	66	28	54	40	40	12	42	28	4	37	11	6	18	18	18	0	34	23	0

^a^ For individuals from mating population:RS1, Rhode Island Red♂×silky♀; SG1, Silky♂×Gushi Chicken♀; RR1-RR5, Rhode Island Red♂×Rhode Island Red♀; SR1 and SR2, silky♂×Rhode Island Red♀.

^b^For tissue: CE, cerebrum; PE, pectoral; VF, visceral fat; HE, heart; KI, kidney; LU, lung; GI, gizzard; PR, proventriculus; TE, testis; CR, crureus; CR1-CR7, crureus from different individuals; Homo1 and Homo2, mixed tissue from different individuals.

^c^ the statistics based on data from 10 tissues of RS1 individuals

^d^ the statistics based on data from all crureus tissues (7 individuals).

^e^ the statistics based on data of all next-generation sequencing samples (18). MAF, minor allele frequency; AAF, alternative allele frequency; UN, undetectable site; TSS, tissue specific heteroplasmic site (MAF≥0.5%).

**Fig 2:**
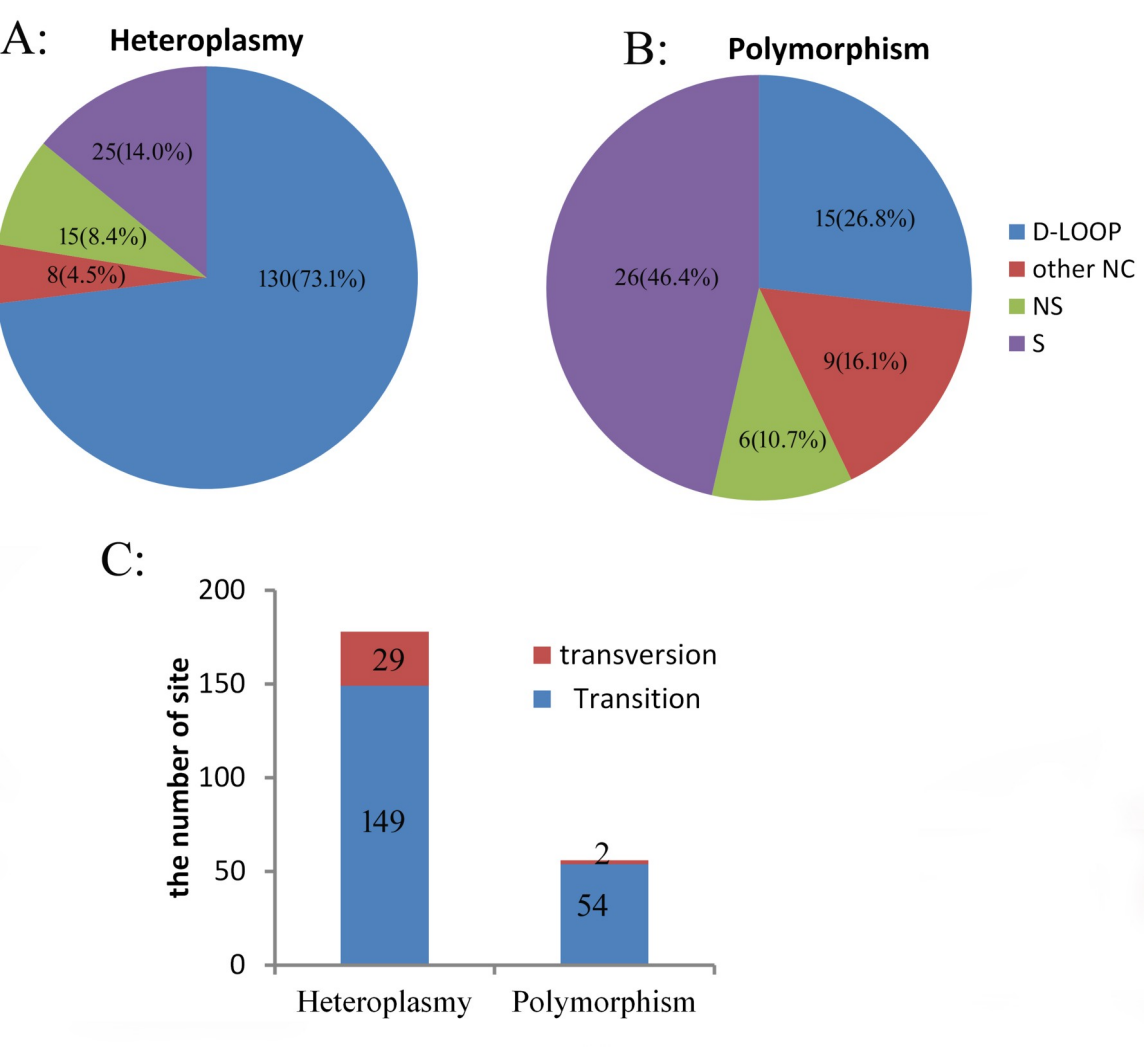
Statistics obtained by next-generation sequencing for heteroplasmy and polymorphisms: A. Heteroplasmy; B. polymorphisms; C. percentages of transitions and transversions.

The D-loop regions comprised about 7% (1227 bp) of the mitochondrial genome, however is the hotspot for mtDNA heteroplasmy in the chicken (Fig 1, Table 3), containing 130 cases (73.1%) of heteroplasmy (Table 3, Fig 2A). On the other hand, the coding regions comprised about 68% (11,390 bp) of the mitochondrial genome, and only 40 cases (22.4%) of heteroplasmy were detected, with 25 synonymous mutations and 15 non-synonymous mutations (Table 3, Fig 2A).

**Table 3:** The distribution of heteroplasmic sites in chicken mitochondrial genome (detected by next-generation sequencing in 18 samples).

Mutation Type^b^	Region	Heteroplasmic site	Polymorphism^a^		Substitution	
			Non- polymorphic	Poly- morphic	Transition	Transversion
NC	D-loop	130	118	12	108	22
	*rRNA*	5	2	3	4	1
	*tRNA*	2	0	2	2	0
	intergene	1	0	1	1	0
	Total	138	120	18	115	23
NS	*COX1*	2	1	1	2	0
	*COX2*	3	2	1	2	1
	*ATP6*	4	4	0	4	0
	*ND5*	2	2	0	0	2
	*CYTB*	1	0	1	1	0
	*ND6*	3	3	0	1	2
	Total	15	12	3	10	5
S	*ND1*	1	0	1	1	0
	*COX1*	3	0	3	3	0
	*COX2*	1	0	1	1	0
	*ATP6*	4	1	3	4	0
	*COX3*	2	0	2	2	0
	*ND3*	2	0	2	2	0
	*ND4L*	1	0	1	1	0
	*ND4*	4	0	4	4	0
	*ND5*	1	0	1	1	0
	*CYTB*	2	1	1	2	0
	*ND6*	4	1	3	3	1
	Total	25	3	22	24	1
Total		178	135	43	149	29

^a^Polymorphic means there are two different predominant alleles among 18 next-generation sequencing samples. Non- polymorphic means there is only one predominant allele among 18 next-generation sequencing samples.

^b^NC, non-coding region; S, Synonymous mutation in the coding region; NS, Non- synonymous mutation in the coding region.

Among the 178 heteroplasmic mutations, most (149) were nucleotide transitions and only 29 mutations were transversions (Fig 2C, Table 3). All transversions were not polymorphic (with the same predominant alleles) and they were detected at low frequencies among the 18 NGS samples, where the predominant reference alleles had AAF values that varied from undetectable to 4.95% (S1 Table).

### Polymorphisms in 18 samples

We detected 56 polymorphic sites (with different predominant alleles) in 18 NGS samples (Table 4, Fig 2B, and S2 Table). Fifteen polymorphic sites were in D-loop regions in the chicken mitochondrial genome. In the coding regions, six polymorphic sites were non-synonymous mutations and 26 polymorphic sites were synonymous mutations (Table 4, Fig 2B). Compared with the distribution of heteroplasmy, less polymorphisms were detected in the same NGS samples and less polymorphisms were located in D-loop regions (*p* < 0.001, Chi-square test). We found that 54 polymorphic sites were transitions and two polymorphic sites were transversions (Table 4, Fig 2C). The ratio of transitions relative to transversions among the polymorphic sites (27:1) was higher (*p* < 0.012, Fisher’s exact test) than that in heteroplasmic sites (5.13:1). The maximum heteroplasmic degree (for MAF) for these polymorphic sites varied among 0.10–40.3% (S2 Table). In total, 43 of the 56 polymorphic sites were heteroplasmic (MAF ≥ 0.5%) and all were transition mutations (Table 4, S2 Table).

**Table 4:** The distribution of polymorphic sites in 18 chicken mitochondrial genome^a^detected by next-generation sequencing.

Mutation Type^b^	Region	Total	Heteroplasmic sites	Substitution	
				Transition	Transversion
NC	D-loop	15	12	15	0
	*rRNA*	5	3	4	1
	*tRNA*	2	2	2	0
	intergene	2	1	2	0
	Total	24	18	23	1
NS	*Cox1*	1	1	1	0
	*Cox2*	2	1	2	0
	*ND4*	1	0	0	1
	*CytB*	2	1	2	0
	Total	6	3	5	1
S	*ND1*	1	1	1	0
	*ND2*	1	0	1	0
	*COX1*	3	3	3	0
	*COX2*	1	1	1	0
	*COX3*	3	2	3	0
	*ATP6*	3	3	3	0
	*ND3*	2	2	2	0
	*ND4L*	1	1	1	0
	*ND4*	5	4	5	0
	*ND5*	2	1	2	0
	*CYTB*	1	1	1	0
	*ND6*	3	3	3	0
	Total	26	22	26	0
Total		56	43(TS)*	54	2

^a^Polymorphic site means there are two different predominant alleles among 18 next-generation sequencing samples.

^b^NC, no-coding region; NS, non-synonymous mutation; S, synonymous mutation. (TS)

*means 43 heteroplasmic sites were transition mutation.

### Heteroplasmic comparison in intra-individual and among individuals

First, the heteroplasmic fluctuation among tissues were investigated based on the NGS data from 10 tissues of the same individual (RS1, offspring of Rhode Island Red♂×Silky♀, aged 60 days). The 141 sites were still heteroplasmic (MAF ≥ 0.5%) in the same individual (Table 2, S1 Table). The heteroplasmic sites varied among the intra-individual tissues. The heteroplasmic sites had allele-specific, position-specific, and tissue-specific features. All of the cases of heteroplasmy had the same predominant allele among the intra-individual tissues (allele-specific, S1 Table). Four sites (*mt*.*A683G*, *mt*.*C737T*, *mt*.*G738A*, and *mt*.*G8682A*) exhibited heteroplasmy in all of the detected tissues (Fig 1, S1 Table). In addition, *mt*.*C737T* (ranged from 9.82% to 40.0%) and *mt*.*G8682A* (ranged from 27.5% to 33.0%) exhibited relative high heteroplasmy across all of the detected tissues (position-specific, S1 Table). In total, 80.8% sites (114 sites) were heteroplasmic in only one tissue (tissue-specific, Fig 1 and S1 Table), 12 sites were heteroplasmic in two tissues, 10 sites were heteroplasmic in three to five tissues, and one site (*mt*.*A16438G*) was heteroplasmic in seven tissues (S1 Table). Each tissue had 10–53 heteroplasmic sites (Table 2). Cases of heteroplasmy were most abundant in skeletal muscles, including crureus (48 sites) and pectoral (53 sites) muscles (Table 2, S1 Table). By contrast, cerebrum, testis, visceral fat, and lung tissues had relatively less heteroplasmic sites. In addition, crureus and cerebrum tissues had less undetectable sites (four sites), and pectoral muscle had the most undetectable sites (66 sites) among intra-individual tissues (Table 2, S1 Table).

Then, the crureus heteroplasmic features were further analyzed based on the NGS data from seven chickens. Among the crureus tissues of seven individuals, 93 sites (MAF≥0.5%) were heteroplasmic (Table 2, S1 Table). The heteroplasmy in crureus tissues varied among individuals, and 56 sites were polymorphic where the maximum MAF varied among 0.1–40.3% (S1 Table). The mitochondrial mutations/heteroplasmy appeared to exhibit clear maternal inheritance features based on the whole chicken mitochondrial genome. The offspring from Rhode Island Red mothers (RR1, RR2, RR3, SR1, and SR2) had the same predominant alleles at all of the heteroplasmic sites. The polymorphic sites were easy to distinguish. We used the polymorphic sites to further infer the inherited features of mutations/heteroplasmy. The SR offspring (SR1 and SR2) had the same predominant allele as the RR offspring (RR1 –RR3) at all 56 polymorphic sites (the mothers of both SR and RR offspring were Rhode Island Red chickens), but their predominant allele were not the same as the RS offspring (the mother of RS1 is Silky) at 42 of 56 polymorphic sites (S1 Table). In addition, the fathers of both the SR offspring (SR1 and SR2) and SG offspring (SG1) were SK, whereas their mothers were Rhode Island Red and Gushi chickens, respectively, and their predominant alleles differed at 44 of 56 polymorphic sites (Table 2).

We observed that the NGS data obtained from two mixed tissues (RR4 and RR5) could effectively reflect the individual heterogeneity at the whole mitochondrial genomic level. RR4 and RR5 shad the same predominant alleles as the other RR individuals (crureus tissues of RR1, RR2, and RR3) at all sites (S1 Table). Twenty cases of heteroplasmy were detected from the RR4 individual, but only four sites (43,713,785,873) was specific to the RR4 individual; whereas 45 cases of heteroplasmy were found in the RR5 individual, and only four heteroplasmic sites (685, 813, 891, and 15435) were specific to the RR5 individual (Table 2, S1 Table).

Two sites (*mt*.*G8682A* and *mt*.*G16121A*) were selected to further validate the accuracy of heteroplasmic data (gained by NGS approach) with PCR-RFLP/Sanger sequencing methods. The *mt*.*G8682A* (D118H) was a heteroplasmic site and its heterogeneity varied from 0.01–33%, whereas *mt*.*G16121A* was a low heterogeneity (maximum heterogeneity 0.80%) and polymorphic site in the detected NGS samples (S1 Table). For the *mt*.*G8682A* site, we first applied Sanger sequencing (S3 Fig) to validate the site heterogeneity with samples where the mt.8682A allele had frequencies of 27.5% (crureus tissue from RS1, S1 Table) and 0.03% (crureus tissue from RR1, S1 Table) in NGS sequencing data. Next, PCR-RFLP was applied to validate the heterogeneity of *mt*.*G8682A* in the NGS samples (S4 Fig). The low heterogeneity individuals (*mt*.*8682A* allele frequency was 0.01–0.15%) according to the NGS approach were detected as homoplasmic *GG* genotype by PCR-RFLP, while 10 tissues from the RS1 individual had high heterogeneity using both NGS (*mt*.*8682A* allele frequency varied among 27.50–33.0%, S3 Table) and PCR-RFLP (*mt*.*8682A* allele varied among 42.97–52.71%, S3 Table). For the *mt*.*G16121A* site, in agreement with the low heterogeneity (*mt*.*16121A* varied among 0–0.80%) determined by NGS approach, all of the samples were detected as homoplasmic *GG/AA* genotype by PCR-RFLP and they had the same predominant alleles identified by NGS approach (S4 Table).

We further investigated the distribution of variation/heterogeneity among breeds at the *mt*.*G8682A* and *mt*.*G16121A* sites with blood DNA. Heteroplasmy appeared to be rare among five breeds at both *mt*.*G8682A* and *mt*.*G16121A* sites, where only one to two heteroplasmic individuals were found by PCR-RFLP (Table 5). For the *mt*.*G8682A* site, homoplasmic *GG* genotypes were predominant in all of the detected breeds, whereas no *AA* genotype individuals were detected and only one heteroplasmic individual (*GA* genotype) was found in SK chickens by PCR-RFLP (Table 5). The *mt*.*G16121A* was polymorphic in the five breeds, where homoplasmic *AA* was the predominant genotype in TB, BY, and SK chickens, homoplasmic *GG* was the predominant genotype in PR chickens, LH individuals had *GG* and *AA* genotypes, and only two heteroplasmic SK individuals (*GA* genotype) were found (Table 5).

**Table 5:** The genotype distribution of *mt*.*G8682A* and *mt*.*G16121A* mutations among breeds (detected by PCR-RFLP in blood DNA).

sites	Genotype	Frequency	LH^a^ (10)	PR (10)	TB (10)	BY (10)	SK (10)
*mt*.*G8682A*	*AA*	0	0.0(0)^b^	0.0(0)	0.0(0)	0.0(0)	0.0(0)
	*GA*	0.02	0.0(0)	0.0(0)	0.0(0)	0.0(0)	0.1(1)
	*GG*	0.98	1(10)	1(10)	1(10)	1(10)	0.9(9)
*mt*.*G16121A*	*AA*	0.66	0.5(5)	0.1(1)	0.9(9)	1(10)	0.8(8)
	*GA*	0.04	0.0(0)	0.0(0)	0.0(0)	0.0(0)	0.2(2)
	*GG*	0.30	0.5(5)	0.9(9)	0.1(1)	0(0)	0.0(0)

^a^LH, White Leghorn; PR, White Plymouth Rock; TB,Tibetan chicken; BY, Beijing You chicken; SK, Silky.

^b^ genotype frequency (Sample number).

### Heterogeneity transmission between generations

Both *mt*.*A5694T* and *mt*.*T5718G* in *mtND2* gene were reported as heteroplasmic in a Gushi chicken resource population [14]. According to NGS data, they were identified as potentially heteroplasmic sites (0.1%≤MAF ≤0.5%), and the maximum heteroplasmy values were 0.26% for *mt*.*A5694T* and 0.33% for *mt*.*T5718G* in the 18 NGS samples (S5 Table). We investigated the heterogeneity of *mt*.*A5694T* and *mt*.*T5718G* in the blood DNA of constructed heteroplasmic population (F0 and F1 generations) by pyrosequencing (Table 6, S6 Table). Both *mt*.*A5694T* and *mt*.*T5718G* were polymorphic in the constructed heteroplasmic population. The *mt*.*5694A* allele (for *mt*.*A5694T*) and the *mt*.*5718T* allele (for *mt*.*T5718G*) were the predominant alleles among individuals and within most individuals. It is interesting that the degree and the occurrence rate of heteroplasmy were significantly higher in F0 chickens (45 weeks old) than F1 population for both the *mt*.*A5694T* and *mt*.*T5718G* sites (Fisher’s exact test, *p* < 0.001). In the F0 population, 15 of 26 individuals were heteroplasmic in Rhode Island Red chickens, and nine to 11 of 24 individuals were heteroplasmic in SK chicken. However, in the F1 population, for the *mt*.*T5718G* site, no cases of heteroplasmy were detected in both RR and RS, and only one to two cases of heteroplasmy were detected in the SS, SR, and SG populations. For the *mt*.*A5694T* site, only one to two cases of heteroplasmy were detected in the RR, RS, SS, SR, and SG populations. These results suggest that the occurrence of heteroplasmy decrease greatly over the generations (Table 6, S6 Table).

**Table 6:** The heteroplasmy distribution of *mt*.*T5718G* and *mt*.*A5694T* in blood DNA of constructed heteroplasmic population (detected by pyrosequencing).

		F0 generation^a^		F1 generation^b^				
population		R	S	RR	RS	SS	SR	SG
Age		45w	45w	60d	60d	60d	60d	60d
Sample number		26	24	27	14	23	33	6
*mt*. *T5718G*	T allele%	59%-100%	3.5%-100%	100%	100%	93%-100%	97%-100%	0–100%
	HE^c^	15	9	0	0	1	1	2
*mt*.*A5694T*	A allele%	66.4%-100%	4.1–100%	97.6%-100%	98.9%-100%	96.1%-100%	98.3%-100%	0–100%
	HE	15	11	2	1	1	1	2

^a^for F0 generation: R, Rhode Island Red; S, Silky.

^b^For F1 generation: RS, Rhode Island Red♂×silky♂; SG, silky♂×Gushi Chicken♀; RR, Rhode Island Red♂×Rhode Island Red♀; SR, silky♂×Rhode Island Red♀; SS, silky♂×silky♂.

^c^HE, the sample number of heteroplasmy (MAF≥0.05%).

We further investigated the heteroplasmic transmission of both the *mt*.*T5718G* and *mt*.*A5694T* sites according to the pedigree information (S7 Table). We found that the occurrence rate and degree of heteroplasmy for both the *mt*.*T5718G* and *mt*.*A5694T* sites decreased dramatically from the F0 to F1 generations (Fisher’s exact test, *p* < 0.0001). For the *mt*.*T5718G* site (S7 Table), 17 of 26 mothers were heteroplasmic (*mt*.*5718T* allele ratio varied among 3.75–97.1%), whereas 52/53 of the offspring from the heteroplasmic mothers were homoplasmic, with *5718T* as the predominant allele. For the *mt*.*A5694T* site (S7 Table), 17 of 26 mothers were heteroplasmic (the *mt*.*5694A* allele ratio varied among 4.5–98.7%), whereas 48/53 of their offspring were homoplasmic, with *mt*.*A5694T* as the predominant allele. In addition, one of the offspring (079–2) was homoplasmic with a *5718T* and *5694A* allele ratio of 100%, whereas its parents had low *mt*.*5718T* and *mt*.*5694A* allele ratios of 3.55–4.5%.

## Discussion

Mitochondrial DNA has been detected in the nuclear genomes of eukaryotes as pseudogenes, or nuclear mitochondrial DNA segments (Numts). Pereira et al. detected at least 13 Numts in the chicken nuclear genome, where the similarity between the Numts and mitochondrial sequences varied from 58.6% to 88.8% [21]. Numts are potential source of contamination in mtDNA research. Thus, it is difficult to filter the nuclear gene sequences that are nearly identical to mtDNA during data analysis [18, 22]. Instead of short-range PCR, single back-to-back LG-PCR can be used for NGS sequencing to reduce the interference from nuclear copies of the mitochondrial genome [22–24].

Our results showed that NGS sequencing was an effective and highly sensitive method for detecting heteroplasmy in the whole chicken mitochondrial genome. We found that heteroplasmy was widespread in the chicken mitochondrial genome where most of the sites exhibited low heteroplasmy. The relatively more common occurrence of heteroplasmy (178) than polymorphisms (56) in the same NGS samples suggests that heteroplasmy can drift to high frequencies within an individual, and eventually be fixed as polymorphisms among individuals. The heteroplasmic positions were not distributed randomly throughout the chicken mtDNA genome (Fig 1). D-loop regions were hotspot regions for the occurrence of heteroplasmy, as also shown in humans [7, 8, 25].

In chickens, heteroplasmy was biased toward transition mutations, as also found in humans, with a higher transition ratio[25–27]. The relatively high ratio of transversions among the low-frequency cases of heteroplasmy may indicate negative selection against heteroplasmy, which suggests that some transversions may be detrimental to mtDNA function, and thus they can only be tolerated at low frequencies and cannot reach “fixation” within an individual.

The occurrence of heteroplasmy varied among tissues within individuals, with allele-specific, position-specific, and tissue-specific features. Heteroplasmy was relatively more common in skeletal muscle, as also found in humans [7, 25, 27]. We found that 81% of the cases of heteroplasmy were present in only one tissue (tissue specific), thereby suggesting that most are probably due to somatic mutations, whereas only a few are likely to be inherited [25].

The *mt*.*G8682A* (D118H) is located in the *Cox2* region and the mutation was predicted to affect the protein’s function (http://sift.bii.a-star.edu.sg/) with a score of 0.02 [28]. Only one individual (RS1) was found to have high heteroplasmy (30.5–33%) in 10 tissues by NGS sequencing and the homoplasmic *AA* genotype was found in none of the samples tested. In addition, only one heteroplasmic individual was detected in five breeds, which suggests that the *mt*.*G8682A* change could be detrimental to the function of mitochondria and that it cannot be fixed. Both *mt*.*A5694T* (T152S) and *mt*.*T5718G* sites (S160A) are transversion mutations in the *mt-ND2* region. The *mt*.*T5718G* mutation was predicted to affect the secondary structure of RNA (http://www.genebee.msu.su/services/rna2_reduced.html). The structure in the *mt*.*5694T*-*mt*.*5718G* haplotype had a higher free energy than that with *mt*.*5694A-mt*.*5718T* (S5 Fig). The reduction in the ratios of the *mt*.*5694T* and *mt*.*5718G* alleles from the F0 generation to the F1 generation in the constructed heteroplasmic population also indicated negative selection against heteroplasmy.

At present, the inheritance of mitochondrial heteroplasmy remains unclear. The central dogma of maternal inheritance for mtDNA remains valid, but Luo et al. reported that mtDNA segregation in some families indicated biparental mtDNA transmission with an autosomal dominant-like inheritance mode [29]. The NGS data obtained in the present study demonstrate that mitochondrial polymorphisms/heteroplasmy in the chicken exhibit maternal inheritance at the entire mitochondrial genomic level. However, our analysis of the heteroplasmic transmission of the *mt*.*A5694T* and *mt*.*T5718G* sites based on pedigree information obtained from the constructed heteroplasmic population by pyrosequencing (Table 6, S7 Table) demonstrated that the intergenerational transmission of mtDNA in heteroplasmic chickens exhibited a rapid shift toward homoplasmy within a single generation. Previously, it was reported that the percentage of heteroplasmic individuals with respect to the *mt*.*A5694T* (same as *mt*.*A5703T* for AP003317) and *mt*.*T5718G* sites (same as *mt*.*T5727G* for AP003317) decreased by approximately 50% from the F0 generation to the F1 generation in a Gushi resource population [14]. In addition, Naue et al. reported a general age dependence for muscle and brain, with a linear correlation in terms of the accumulation of heteroplasmy in muscle [27], which could explain the high heteroplasmy in F0 individuals.

Mitochondria undergo a bottleneck during oogenesis, so it is expected that the frequency of alleles at heteroplasmic sites will differ even among related individuals [30]. However, in agreement with our results, Wai et al. found that intergenerational mtDNA transmission in heteroplasmic mice exhibited a rapid shift toward homoplasmy within a single generation[31, 32]. Guo et al. showed that very low heteroplasmy variants (down to almost 0.1%) in humans are inherited maternally and that this inheritance decreased to about 0.5%[13]. Rebolledo-Jaramillo et al. observed dramatic shifts in the frequency of heteroplasmy between generations and estimated the effective size of the germline mtDNA bottleneck at only ∼30–35 [33].

In conclusion, NGS data showed that mtDNA heteroplasmy was widespread in the chicken mitochondrial genome, where most cases of heteroplasmy had a low ratio. D-loop regions were identified as hotspots for heteroplasmy. Heteroplasmy was biased toward transition mutations, but the ratio of transversions relative to transitions in heteroplasmic sites was higher than that in polymorphic sites. Intergenerational mtDNA transmission in heteroplasmic chickens exhibited a rapid shift toward homoplasmy within a single generation according to analyses of heteroplasmy at *mt*.*A5694T* and *mt*.*T5718G*. Our findings suggest that heteroplasmy may be under negative selection to some degree in the chicken.

## Supporting information

S1 Fig Products obtained by long-range PCR. M. DNA markers: PCR products 1–4.
(TIF)

S2 Fig Depth of NGS sequencing. (TIF)

S3 Fig Validation of *mt*.*G8682A* heteroplasmy by Sanger sequencing: A. 27.5% for *mt*.*8682A* allele by NGS sequencing; B. 0.03% for mt.8682A allele by NGS sequencing.
(TIF)

S4 Fig Genotypes of *mt.G8682A* and *mt.G16121A* detected by PCR-RFLP. PCR products were cut using *Hinf*I. *GA* denotes heteroplasmic individuals. *AA* and *GG* denote homoplasmic individuals with only allele A or G. A. *mt*.*G8682A* site sequence with mt.8682G cut into 196-bp and 36-bp fragments (36-bp fragment was not detected by gel electrophoresis). B. *mt*.*G16121A* site sequence with mt.16121A cut into 145-bp and 31-bp fragments (31-bp fragment was not detected by gel electrophoresis).
(TIF)

S5 Fig Predicted RNA secondary structures for the sequences containing the *mt.A5694T* and *mt.T5718G* changes. (TIF)

S1 Table The information of heteroplasmic sites detected by NGS sequencing from 18 samples (minor allele frequency≥0.5%). (XLS)

S2 Table The information of polymorphic sites among 18 samples detected by NGS sequencing. (XLSX)

S3 Table The comparison of *mt.G8682A* heteroplasmy detected by PCR—RFLP and NGS sequencing. (DOCX)

S4 Table The comparison of mt.G16121A heteroplasmy detected by NGS sequencing and PCR–RFLP. (DOCX)

S5 Table The information of heteroplasmic sites detected by NGS sequencing from 18 samples in mtND2 region (minor allele frequency≥0.1%). (XLSX)

S6 Table The heteroplasmic information of *mt.A5694T* and *mt.T5718G* in bood of DNA of constructed heteroplasmic population(detected by pyrosequencing). (XLSX)

S7 Table The Heteroplasmic transmission of *mt.T5718G* and *mt.A5694T* between F0 and F1 generations of the constructed heteroplasmic population(detected by pyrosequencing from the bood DNA). (XLSX)
