# Supplementary figures and images for: Heteroplasmy in the complete chicken mitochondrial genome
Source: PLoS One. 2019 Nov 8;14(11):e0224677. doi: 10.1371/journal.pone.0224677 (PMC6839896; doi:10.1371/journal.pone.0224677)

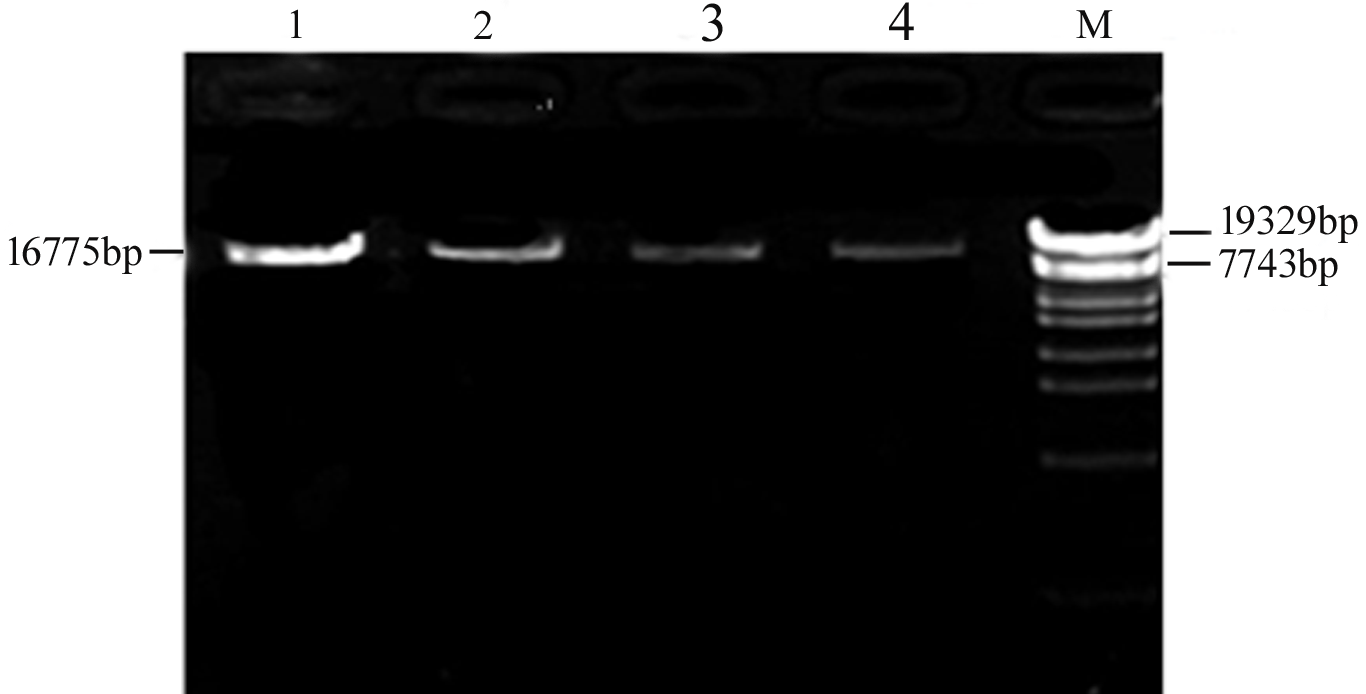

Supplement: S1 Fig — M. DNA markers: PCR products 1–4. (TIF) [file pone.0224677.s001.tif]

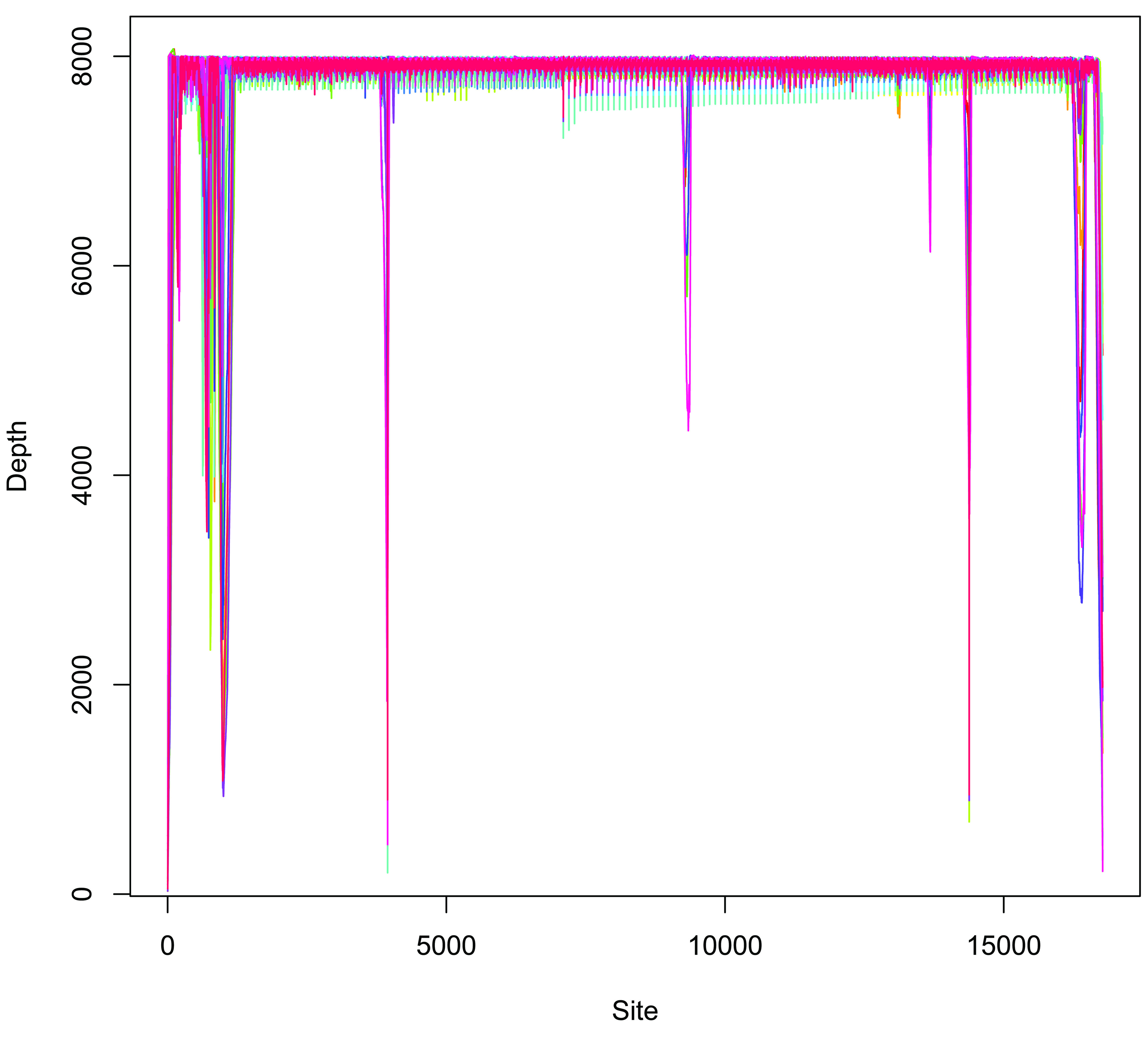

Supplement: S2 Fig — (TIF) [file pone.0224677.s002.tif]

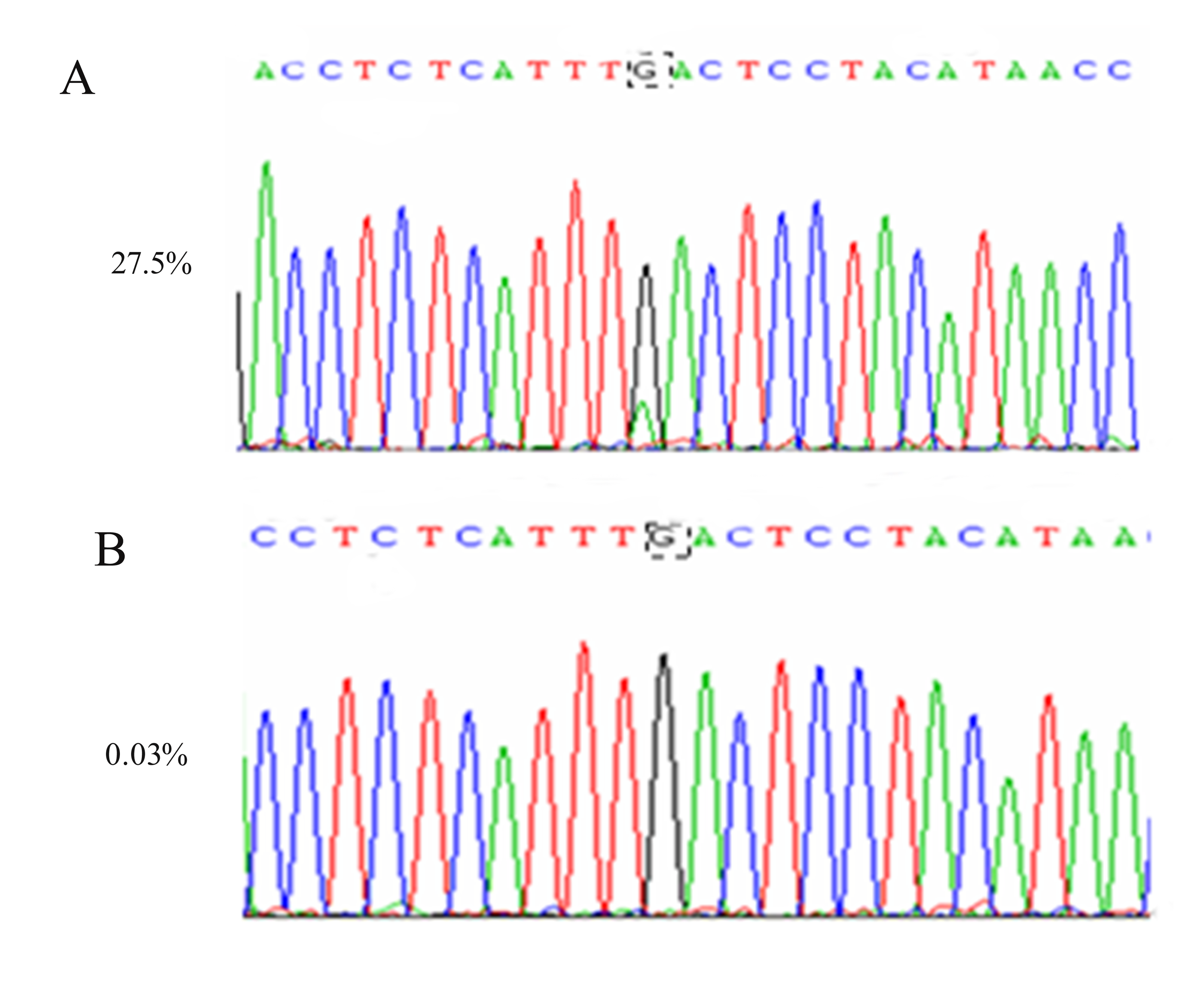

Supplement: S3 Fig — Validation of mt.G8682A heteroplasmy by Sanger sequencing: A. 27.5% for mt.8682A allele by NGS sequencing; B. 0.03% for mt.8682A allele by NGS sequencing. (TIF) [file pone.0224677.s003.tif]

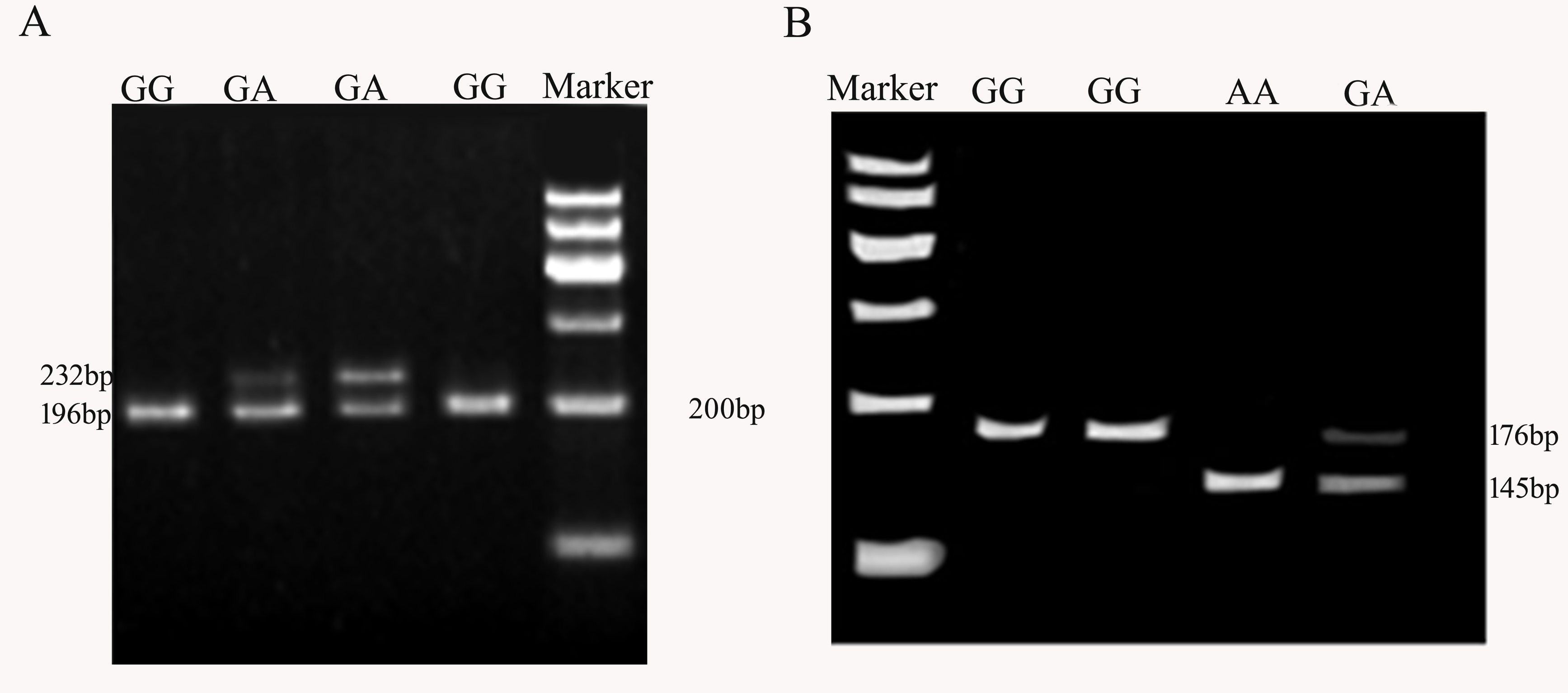

Supplement: S4 Fig — PCR products were cut using HinfI. GA denotes heteroplasmic individuals. AA and GG denote homoplasmic individuals with only allele A or G. A. mt.G8682A site sequence with mt.8682G cut into 196-bp and 36-bp fragments (36-bp fragment was not detected by gel electrophoresis). B. mt.G16121A site sequence with mt.16121A cut into 145-bp and 31-bp fragments (31-bp fragment was not detected by gel electrophoresis). (TIF) [file pone.0224677.s004.tif]

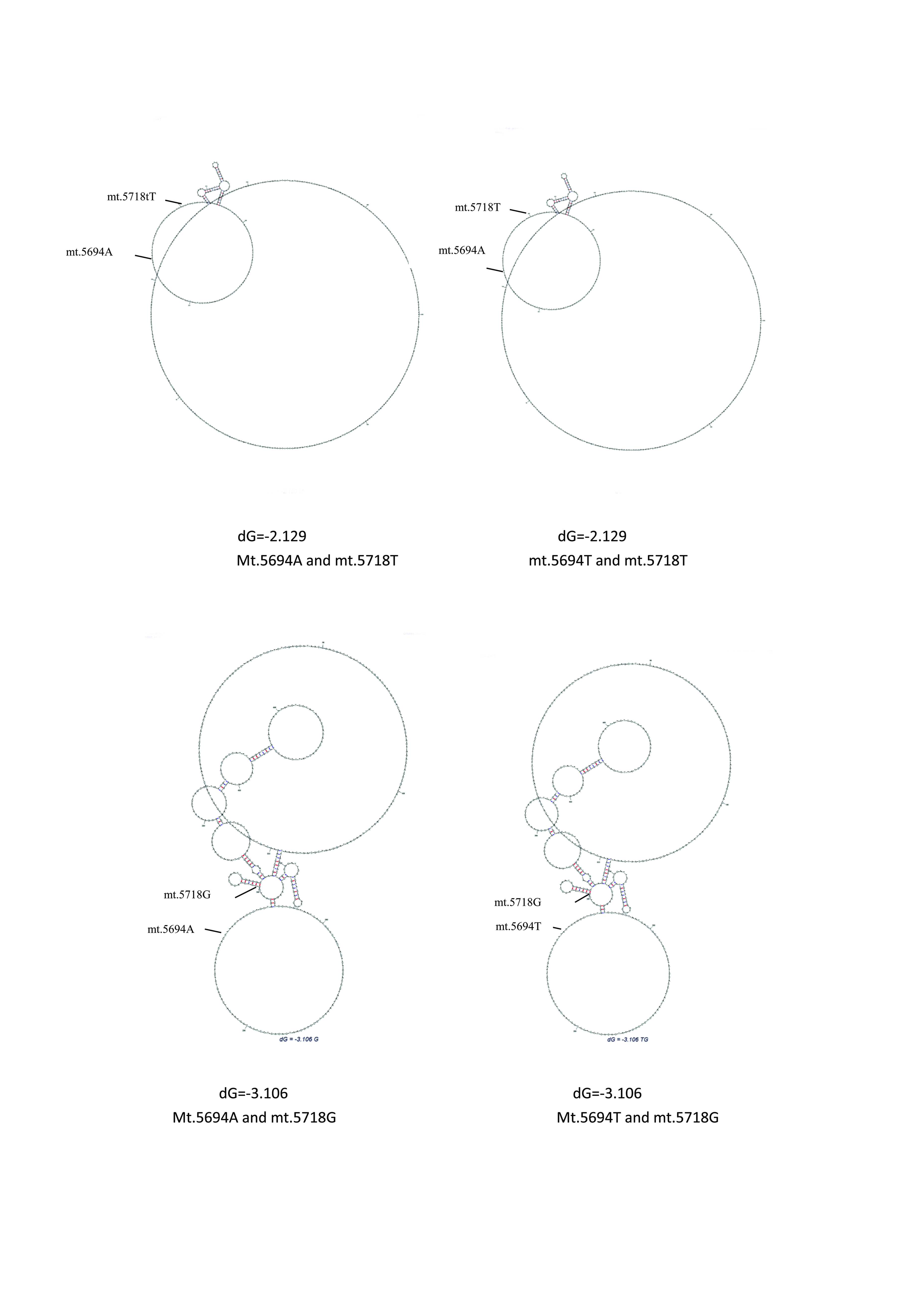

Supplement: S5 Fig — (TIF) [file pone.0224677.s005.tif]
