# Supplementary material for: Heteroplasmy in the complete chicken mitochondrial genome
Source: PLoS One. 2019 Nov 8;14(11):e0224677. doi: 10.1371/journal.pone.0224677 (PMC6839896; doi:10.1371/journal.pone.0224677)
Supplement: S3 Table — (DOCX) [file pone.0224677.s008.docx]

**Supplementary Table 3 The comparison of *mt.G8682A* heteroplasmy detected by PCR - RFLP and NGS sequencing^a^**

| Sample |  | PCR-RFLP | | |  | NGS sequencing | | | |
| --- | --- | --- | --- | --- | --- | --- | --- | --- | --- |
| name^b^ | Tissue^c^ | G（%） | A（%） | Genotype |  | G（%） | | A（%） | |
| RR5 | Homo2 | 100 | 0 | GG | 99.85 | | 0.15 | |  |
| RR4 | Homo1 | 100 | 0 | GG | 99.94 | | 0.06 | |  |
| SR2 | CR7 | 100 | 0 | GG | 99.99 | | 0.01 | |  |
| SR1 | CR6 | 100 | 0 | GG | 99.97 | | 0.03 | |  |
| RR3 | CR5 | 100 | 0 | GG | 99.95 | | 0.05 | |  |
| RR2 | CR4 | 100 | 0 | GG | 99.95 | | 0.05 | |  |
| RR1 | CR3 | 100 | 0 | GG | 99.97 | | 0.03 | |  |
| SG1 | CR2 | 100 | 0 | GG | 99.96 | | 0.04 | |  |
| RS1 | CR1 | 57.03 | 42.97 | GA | 72.50 | | 27.50 | |  |
| RS1 | PE | 55.51 | 44.49 | GA | 69.50 | | 30.50 | |  |
| RS1 | PR | 53.65 | 46.35 | GA | 68.70 | | 31.30 | |  |
| RS1 | KI | 47.29 | 52.71 | GA | 67.00 | | 33.00 | |  |
| RS1 | TE | 47.86 | 52.14 | GA | 68.10 | | 31.90 | |  |
| RS1 | VF | 48.26 | 51.74 | GA | 68.90 | | 31.10 | |  |
| RS1 | HE | 51.80 | 48.20 | GA | 68.20 | | 31.80 | |  |
| RS1 | LU | 55.64 | 44.36 | GA | 69.20 | | 30.80 | |  |
| RS1 | GI | 49.81 | 50.19 | GA | 68.10 | | 31.90 | |  |
| RS1 | CE | 48.43 | 51.57 | GA | 68.30 | | 31.60 | |  |

^a^PCR-RFLP, PCR-restriction fragment length polymorphism; NGS, next-generation sequencing. ^b^For sample from mating population: RR1-5, Rhode Island Red♂× Rhode Island Red♀; SR1 and SR2, silky♂×Rhode Island Red♀; RS1, Rhode Island Red♂×silky♀; SG1, silky♂×Gushi Chicken♀. ^c^For tissues: CR, crureus; PE, pectoral; PR, proventriculus; KI, kidney; VF, visceral fat; HE, heart; TE, testis; LU, lung; GI, gizzard; CE, cerebrum; Homo, mixed tissue
