# Supplementary material for: Heteroplasmy in the complete chicken mitochondrial genome
Source: PLoS One. 2019 Nov 8;14(11):e0224677. doi: 10.1371/journal.pone.0224677 (PMC6839896; doi:10.1371/journal.pone.0224677)
Supplement: S4 Table — (DOCX) [file pone.0224677.s009.docx]

**Supplementary Table 3**. **The comparison of *mt.G16121A* heteroplasmy detected by NGS sequencing and PCR - RFLP**

|  |  | PCR-RFLP | |  | |  | | NGS sequencing | | | | | | |  |
| --- | --- | --- | --- | --- | --- | --- | --- | --- | --- | --- | --- | --- | --- | --- | --- |
| Sample^a^ | Tissue^b^ | G（%） | A（%） | Genotype | |  | |  | G（%） | | | A（%） | | | |
| RR5 | Homo | 100 | 0 | GG | | | 99.57 | | | | 0.43 | |  |  |  |
| RR4 | Homo | 100 | 0 | GG | | | 99.78 | | | | 0.22 | |  |  |  |
| SR2 | CR7 | 100 | 0 | GG | | | 99.95 | | | | 0.05 | |  |  |  |
| SR1 | CR6 | 100 | 0 | GG | | | 99.94 | | | | 0.06 | |  |  |  |
| RR3 | CR5 | 100 | 0 | GG | | | 99.86 | | | | 0.14 | |  |  |  |
| RR2 | CR4 | 100 | 0 | GG | | | 99.84 | | | | 0.16 | |  |  |  |
| RR1 | CR3 | 100 | 0 | GG | | | 99.84 | | | | 0.16 | |  |  |  |
| SG1 | CR2 | 0 | 100 | AA | | | 0 | | | | 100 | |  |  |  |
| RS1 | CR1 | 0 | 100 | AA | | | 0.30 | | | | 99.70 | |  |  |  |
| RS1 | PE | 0 | 100 | AA | | | 0.80 | | | | 99.20 | |  |  |  |
| RS1 | PR | 0 | 100 | AA | | | 0.10 | | | | 99.90 | |  |  |  |
| RS1 | KI | 0 | 100 | AA | | | 0 | | | | 100 | |  |  |  |
| RS1 | TE | 0 | 100 | AA | | | 0 | | | | 100 | |  |  |  |
| RS1 | VF | 0 | 100 | AA | | | 0.10 | | | | 99.90 | |  |  |  |
| RS1 | HE | 0 | 100 | AA | | | 0.20 | | | | 99.80 | |  |  |  |
| RS1 | LU | 0 | 100 | AA | | | 0 | | | | 100 | |  |  |  |
| RS1 | GI | 0 | 100 | AA | | | 0.30 | | | | 99.70 | |  |  |  |
| RS1 | CE | 0 | 100 | AA | | | 0.20 | | | | 99.80 | |  |  |  |
|  |  | | | |  | | | | |  | | | |  |  |

^a^For sample from mating population: RR1-5, Rhode Island Red♂× Rhode Island Red♀; SR1 and SR2, silky♂×Rhode Island Red♀; RS1, Rhode Island Red♂×silky♀; SG1, silky♂×Gushi Chicken♀. ^b^For tissues: CR, crureus; PE, pectoral; PR, proventriculus; KI, kidney; TE, testis; VF, visceral fat; HE, heart; LU, lung; GI, gizzard; CE, cerebrum; Homo, mixed tissue.
